# Supplementary material for: Variation in the diversity and richness of parasitoid wasps based on sampling effort
Source: PeerJ. 2018 Apr 5;6:e4642. doi: 10.7717/peerj.4642 (PMC5889912; doi:10.7717/peerj.4642)
Supplement: Supplemental Information 1 [file peerj-06-4642-s001.docx]

**Supplementary Information 1 – Molecular Information**

To improve the accuracy of morphospecies identifications, a total of 90 Ichneumonidae specimens were used for genetic analysis. Not all specimens were submitted for molecular analysis. Males and females of some specimens were able to be identified to the species level via their external morphology. For molecular analysis two females and two males were selected from each morphospecies, but this was not always possible as some morphospecies were singletons.

DNA barcodes were obtained using DNA extracts prepared from removal of a single hind leg of the specimen, and using a glass fibre protocol (Ivanova *et al*. 2006). Extracts were re-suspended in 30 μl of dH2O, and a 658-bp region near the 5’ terminus of the COI gene was amplified using standard primers LepR1 (TAAACTTCTGGATGTCCAAAAAATCA) and LepF1 (ATTCAACCAATCATAAAGAT-ATTGG) following established protocols (Smith et al. 2006).

Confirmation of morphospecies delimitation was obtained using a neighbour-joining tree using Kimura-2-parameter distances and aligned using MUSCLE within the BOLD platform.

The Barcode Index Number (BIN) system (Ratnasingham & Hebert 2013) was utilised to match females and males. All sequences are publicly available on BOLD (http://www.boldsystems.org) under the project “IchneumonidaeDiversityMSc (LKMSC)” with Process Id numbers from LKMSC191-16 to LKMSC285-16.

**References**

Ivanova, N. V., de Waard, J. R. & Hebert, P. D. N. (2006). An inexpensive, automation-friendly protocol for recovering high-quality DNA .*Molecular Ecology Notes, 6*, 998–1002.

Ratnasingham, S., & Hebert, P. D. N. (2013). A DNA-based registry for all animal species: The Barcode Index Number (BIN) system. *PLoS ONE*, *8*(7), e66213.

Smith, M. A., Woodley, N. E., Janzen, D. H., Hallwachs, W., & Hebert, P. D. (2006). DNA barcodes reveal cryptic host-specificity within the presumed polyphagous members of a genus of parasitoid flies (Diptera: Tachinidae). *Proceedings of the National Academy of Sciences of the United States of America*, *103*(10), 3657–3662.
